# Vegetal Undercurrents—Obscured Riverine Dynamics of Plant Debris

**DOI:** 10.1029/2021JG006726

**Published:** 2022-03-28

**Authors:** Melissa S. Schwab, Robert G. Hilton, Negar Haghipour, J. Jotautas Baronas, Timothy I. Eglinton

**Affiliations:** ^1^ Department of Earth Sciences ETH Zurich Zurich Switzerland; ^2^ Now at Jet Propulsion Laboratory California Institute of Technology Pasadena CA USA; ^3^ Department of Earth Sciences University of Oxford Oxford UK; ^4^ Laboratory of Ion Beam Physics ETH Zurich Zurich Switzerland; ^5^ Institut de Physique du Globe de Paris Université de Paris Paris France

**Keywords:** organic carbon, plant biomarker, radiocarbon, Arctic, suspended sediment, undercurrent

## Abstract

Much attention has been focused on fine‐grained sediments carried as suspended load in rivers due to their potential to transport, disperse, and preserve organic carbon (OC), while the transfer and fate of OC associated with coarser‐grained sediments in fluvial systems have been less extensively studied. Here, sedimentological, geochemical, and biomolecular characteristics of sediments from river depth profiles reveal distinct hydrodynamic behavior for different pools of OC within the Mackenzie River system. Higher radiocarbon (^14^C) contents, low N/OC ratios, and elevated plant‐derived biomarker loadings suggest a systematic transport of submerged vascular plant debris above the active riverbed in large channels both upstream of and within the delta. Subzero temperatures hinder OC degradation promoting the accumulation and waterlogging of plant detritus within the watershed. Once entrained into a channel, sustained flow strength and buoyancy prevent plant debris from settling and keep it suspended in the water column above the riverbed. Helical flow motions within meandering river segments concentrate lithogenic and organic debris near the inner river bends forming a sediment‐laden plume. Moving offshore, we observe a lack of discrete, particulate OC in continental shelf sediments, suggesting preferential trapping of coarse debris within deltaic and neritic environments. The delivery of waterlogged plant detritus transport and high sediment loads during the spring flood may reduce oxygen exposure times and microbial decomposition, leading to enhanced sequestration of biospheric OC. Undercurrents enriched in coarse, relatively fresh plant fragments appear to be reoccurring features, highlighting a poorly understood yet significant mechanism operating within the terrestrial carbon cycle.

## Graphical abstract

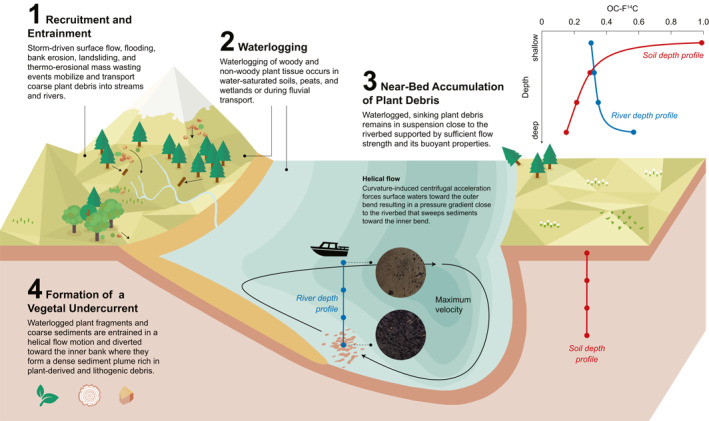

**Key Points**

- ^14^C content of the suspended load increases toward the riverbed corresponding to enrichment in discrete, plant‐derived organic carbon (OC)
- Accumulation of coarse OC is supported by high bulk and plant‐derived biomarker loadings
- Reoccurring nature of these observations suggests in‐river transport of submerged plant debris is a systematic and important phenomenon

## 1 Introduction

Rivers, termed the “arteries” of the planet, are vital links in the global carbon cycle, connecting terrestrial and marine carbon reservoirs and delivering up to 20 Gt yr^−1^ of sediment (Milliman & Farnsworth, 2013) and ca. 0.2 GtC yr^−1^ of particulate organic carbon (POC) to continental margins (Galy et al., 2015; Seitzinger et al., 2005). They also act to integrate signals emanating from their drainage basins and provide an effective means to assess regional‐scale impacts of environmental change on biogeochemical cycles (T. I. Eglinton et al., 2021). Terrestrially derived OC can be subject to extensive remineralization and modification during mobilization, transport, and storage within fluvial systems (Battin et al., 2009; Blair & Aller, 2012; Ward et al., 2017), associated with globally important greenhouse gas emissions from river surfaces (Lauerwald et al., 2015; Raymond et al., 2013). However, on long (>10^4^ yr) timescales, the export and burial of biospheric POC in marine sediments result in a net transfer of atmospheric carbon to the sedimentary reservoir, mitigating CO_2_ levels in the atmosphere (Bianchi et al., 2018; Burdige, 2005; Galy et al., 2015; Hilton et al., 2015; Leithold et al., 2016).

Particulate organic matter entrained and carried in rivers can be heterogeneous in its source, chemical composition, and size, and subjected to hydrodynamic processes that affect its dispersal, reactivity, and age (Bianchi et al., 2018; Freymond, Kündig et al., 2018; Repasch et al., 2022; Ward et al., 2017; Yu et al., 2019). River depth profiles typically show variations in suspended sediment concentration and grain size as a function of depth (Bouchez, Gaillardet et al., 2011; Bouchez, Métivier et al., 2011; Galy, France‐Lanord, & Lartiges, 2008). Hydrodynamic sorting by particle size, shape, and density introduces a vertical distribution of the suspended load causing coarser, denser particles to be transported near the riverbed, whereas fine‐grained sediments are more homogeneously dispersed throughout the water column (Bouchez, Lupker et al., 2011; Bouchez, Métivier et al., 2011; Lupker et al., 2011). Coarse, lithogenic particles derived from weathered sedimentary bedrock are often enriched in ^14^C‐free, petrogenic OC (Blair et al., 2004; Bouchez et al., 2010; Hedges, 1992). In contrast, silt‐ and clay‐sized particles are often associated with biospheric organic matter due to physicochemical interactions between OC and the mineral matrix (e.g., Blair & Aller, 2012; Hemingway et al., 2019; Keil et al., 1994).

Hydrodynamic sorting further influences the OC dispersal from land to adjacent shelf regions (Bao, Uchida et al., 2018; Bao, Zhao et al., 2019; Bröder et al., 2018; Bröder, Tesi, Salvadó et al., 2016; Tesi et al., 2016). In general, coarse sediments and discrete plant‐derived debris are often retained in deltaic environments, while fine‐grained, mineral‐associated OC is preferentially transported offshore (Bao, Blattmann et al., 2019; Bao, van der Voort et al., 2018; Bianchi et al., 2007; Keil et al., 1994; X. Sun et al., 2021). In addition to hydrodynamic controls, the efficiency of OC export and burial associated with fine‐grained sediments is generally considered greater due to a larger mineral surface area (SA) that promotes stabilization of associated organic matter (Bao, Zhao et al., 2019; Kögel‐Knabner et al., 2008; Mayer, 1994; Tesi et al., 2016; Wakeham & Canuel, 2016). Sorption, aggregation, or occlusion create protective environments rendering OC inaccessible to microorganisms and extracellular enzymes enhancing the resistance of organic matter to degradation (Hedges & Keil, 1995; Keil & Mayer, 2014; Mayer, 1994; Zonneveld et al., 2010). In contrast, sand‐sized material is often regarded as carbon‐poor due to lower availability of mineral SA for binding and higher oxygen permeability that favors OC remineralization (Burdige, 2005, 2007). However, recent studies have reported accumulation and efficient burial of abundant woody and non‐woody tissue in sandy turbiditic successions (Hage et al., 2020; Lee et al., 2019; Leithold et al., 2016; Sparkes et al., 2015). Rapid transport and burial of plant biomass induced by hyperpycnal flows reduce OC degradation, allowing for enhanced preservation in coarse siliciclastic sediments (Hage et al., 2020; Kao et al., 2014; Lee et al., 2019). The transport and fate of organic matter in fluvial water columns may therefore be more complex than hitherto considered, yet relatively few studies have examined hydrodynamic controls on biospheric OC dynamics (Bouchez et al., 2014; Freymond, Lupker et al., 2018; Galy, Beyssac et al., 2008).

The Mackenzie River annually discharges about 128 Mt yr^−1^ of sediment to the coastal margin, rendering it the largest sediment supplier to the Arctic Ocean (Carson et al., 1999; Holmes, 2002; Macdonald et al., 1998). Its hydrograph is strongly impacted by the ice break‐up and snowmelt, forcing the majority of sediment and freshwater to be delivered within a few months associated with the spring freshet (Hill et al., 2001). While one‐third of the sediment load is estimated to be trapped within the Mackenzie Delta, which consists of numerous lakes and channels, the remainder is deposited on the continental shelf (Carson et al., 1998; Emmerton et al., 2007; Macdonald et al., 1998). These fluvial sediments contain approximately 2.2 Mt yr^−1^ of POC which is largely derived from eroding soils in permafrost zones that experienced severe pre‐aging prior to fluvial export (Goñi et al., 2005; Guo et al., 2007; Hilton et al., 2015; Macdonald et al., 1998; Vonk et al., 2015, 2016). A recent study by Vonk et al. (2019) identified a significant pool of years to decades‐old biospheric OC mobilized during the spring freshet, suggesting the active entrainment of litter and surface soil material. Depending on their physical properties, mobilization patterns of biospheric carbon can differ considerably. Runoff exerts the dominant control on the delivery of fresh organic matter from surface sources, in analogy with other river systems around the world (Hilton, 2017), whereas mineral‐bound OC is primarily derived from thermal and mechanical erosion (Feng et al., 2015; Feng, Vonk et al., 2013). However, our knowledge is still limited with respect to hydrodynamic processes that impact the dispersion and export of organic matter once entrained into a fluvial network.

In this study, we combine physical (grain size, SA), bulk (%OC, N/OC, OC‐F^14^C), and molecular (vascular plant biomarker) organic geochemical properties of suspended load samples collected at different locations and water column depths to examine in‐river transport of particulate OC in the Mackenzie River basin. Our measurements reveal an enrichment of discrete biospheric debris entrained in the suspended load near the riverbed. We explore potential hydrodynamic mechanisms which promote the formation and sustainment of waterlogged, plant debris‐laden undercurrents. We assess implications for the fate of biospheric OC along the land to ocean continuum, for the significance of plant‐derived biomass in regional carbon budgets, and with respect to impending changes in organic matter export in response to continued climate warming.

## 2 Methods

### 2.1 Sample Collection

**Figure 1:**
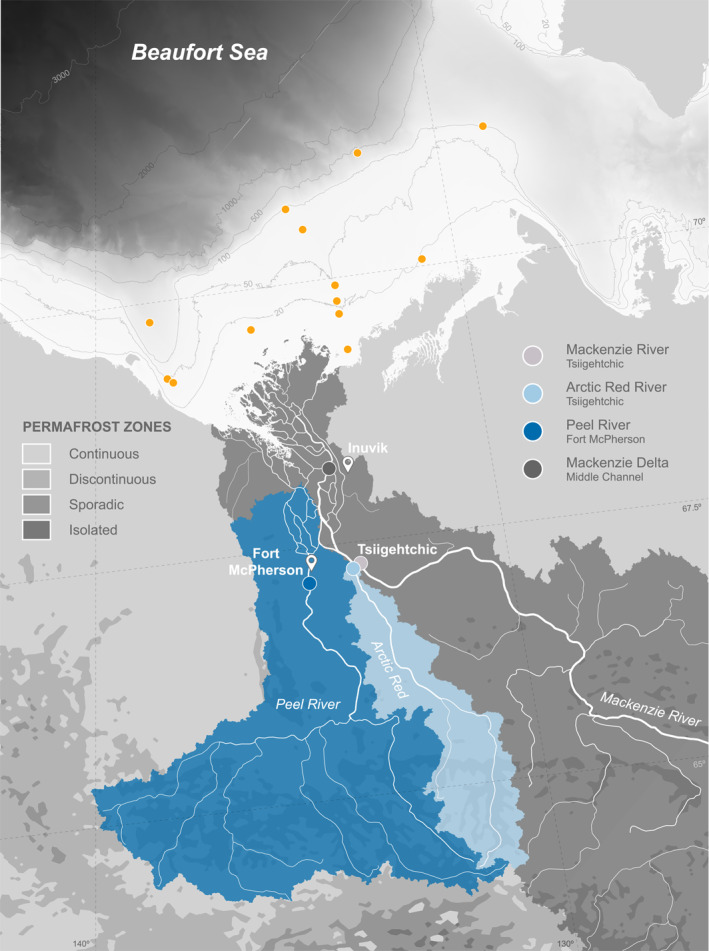
Locations of river depth profiles along the Mackenzie River at Tsiigehtchic (light gray) and the delta (dark gray) complemented by the major tributaries, the Arctic Red (light blue), and the Peel Rivers (dark blue). Yellow dots denote published surface and core sample locations on the Mackenzie Shelf (Drenzek et al., 2007; Goñi et al., 2005; Goñi, O’Connor et al., 2013; Hilton et al., 2015; Vonk et al., 2015). Permafrost zone coverage is obtained from Obu et al. (2019). The gray shaded area shows the International Bathymetric Chart of the Arctic Ocean (IBCAO, Version 4.1) (Jakobsson et al., 2020).

The study area is situated in the northern Mackenzie River basin (Figure 1). Suspended sediments were collected from four locations, comprising the Arctic Red River, the Peel River at Fort McPherson, the Mackenzie River at Tsiigehtchic near the apex of the delta before the confluence with the Arctic Red River and the largest channel within the Mackenzie Delta (Middle Channel), during peak freshet/receding water stages in July 2013, June 2017, 2018, and 2019 (Hilton et al., 2015; Schwab et al., 2020). We also use previously published data from May/June 2011 and September 2010 from Hilton et al. (2015). Several transects were recorded by crossing the river normal to the flow, measuring channel depth and water velocity using Acoustic Doppler Current Profiling (ADCP; RioGrande, RD Instruments, 600 kHz in 2017–2018; RiverRay, RD Instruments, 600 kHz in 2019) mounted to the side of a boat. Sampling locations were identified based on the maximum channel depth and the highest backscatter (corresponding to the highest turbidity). To account for hydrodynamic transport and sorting of suspended particles within the river (Bouchez, Gaillardet et al., 2011; Bouchez, Métivier et al., 2011), we recovered water from different depths using a modified, horizontally mounted ∼5.1 L Niskin bottle (Hilton et al., 2015). The collected water was transferred to sterilized plastic bags (Jigsaw Bag in Box Ltd) and weighed to obtain the sample volume. Within 48 h, the samples were filtered through Millipore polyethersulfone filters (PES; ∅ 142 mm, 0.22 μm) using pre‐cleaned Teflon filtration units. Sediment‐laden filters (*n* = 84) were folded, wrapped in pre‐combusted aluminum foil envelopes, and immediately frozen. In addition to suspended sediment samples, bed material (*n* = 6) was dredged at the base of the depth transect using a metal bucket and decanted into sterile bags. Freshly deposited riverbank sediments (*n* = 21) were collected adjacent to the channel at several locations (Table S1).

### 2.2 Sedimentological Analyses

Prior to particle size and SA analyses, OC was removed from freeze‐dried samples by thermal oxidation (450°C, 6 h). Grain size distributions were measured using a Malvern Mastersizer 2000 laser diffraction granulometer (Malvern Instruments Ltd) coupled to a Hydro 2000S ultrasonic dispersion unit. Analytical reproducibility better than 10% was estimated based on the relative standard deviation of the modal size of repeated measurements. Surface area analyses were performed on a NOVA 4000e Surface Area Analyzer with N as adsorbing gas. Degassed samples (350°C, 8 h; Quantachrome FLOVAC degasser) were analyzed using the Quantachrome NovaWin software generating a 5‐point BET method (Brunauer et al., 1938).

### 2.3 Elemental and Isotopic Analyses of Organic Carbon

Freeze‐dried sediments were ground and homogenized. A known mass (∼30 mg) of sample material (corresponding to ∼200–300 μgC) was placed in Ag capsules and fumigated with HCl vapor (70°C, 72 h) removing inorganic carbon (Bao, McNichol et al., 2018), followed by neutralization with NaOH (70°C, 72 h). Vapor‐acid treated samples were wrapped in tin boats and analyzed for N and OC contents on an Elementar Vario MICROcube elemental analyzer at the Laboratory of Ion Beam Physics (ETH Zurich). The ^14^C activity was measured directly as CO_2_ on a Mini Carbon Dating System (MICADAS, Ionplus AG; ETH Zurich; McIntyre et al., 2016; Wacker et al., 2013). Samples were calibrated against Oxalic Acid II (NIST SRM 4990C) and corrected for a full procedural blank (of ∼3–5 μgC) using a set of in‐house soil and shale standards as per Haghipour et al. (2019) and reported as fraction modern, F^14^C (Reimer et al., 2004).

### 2.4 Lipid Extraction, Separation, and Quantification

Total lipid extracts of pre‐weighed sediment samples from the 2017 campaign were extracted using a Microwave Accelerated Reaction System (MARS, CEM Corporation) with dichloromethane/methanol (DCM/MeOH 9:1) at 100°C for 25 min. Corresponding total lipid extracts of samples collected in 2018 and 2019 were obtained using an EDGE automated extraction system (CEM Corporation) and three extraction‐rinse cycles with a DCM/MeOH 9:1 solvent mixture (100°C, 5 min). Extracted lipids were then saponified at 70°C for 2 h using 10 mL of 0.5 M KOH in MeOH. After adding 10 mL MilliQ with NaCl, the neutral fraction was recovered by liquid/liquid extraction with hexane. The remaining extract was acidified with concentrated HCl to pH 2 and back‐extracted with hexane:DCM (4:1) recovering the acid fraction. Apolar components of the neutral fraction were eluted with 4 mL hexane using Pasteur pipette columns containing 1% deactivated SiO_2_ and Na_2_SO_4_. Prior to the purification of the acid fraction, fatty acids were methylated to fatty acid methyl esters (FAMEs) with MeOH/HCl (95:5, 70°C, 12 hr). FAMEs were separated via elution with hexane:DCM 3:7 and DCM. Concentrations of *n*‐alkanes and *n*‐fatty acids were determined on a gas chromatograph with a flame ionization detector (GC‐FID, Agilent Technologies 7890A) equipped with an Agilent VF‐1 ms column (30 m × 320 μm ID × 0.25 μm film thickness). The temperature program of a total of 35.2 min starts with a 1 min hold time at 50°C and ramps to 320°C at a 10°C min^−1^ rate with an isothermal hold time of 5 min at 320°C. Samples were calibrated to an external standard run at 3–5 dilutions between sample measurements. We divided dry sediment‐normalized *n*‐alkane (ΣC_25–35_), short‐chain (ΣC_16–18_), and long‐chain (ΣC_24–32_) *n*‐fatty acid concentrations (μg g^−1^ dry sediment) by their respective SA (m^2^ g^−1^) to obtain “biomarker loadings” in μg m^−2^.

*n*‐Alkanes derived from fresh vascular plants typically display an odd‐over‐even predominance for long‐chain (*n*‐C_25_+) homologs (Bray & Evans, 1961; G. Eglinton & Hamilton, 1967), whereas even homologs are often associated with biological or thermal degradation of organic matter (Zhou et al., 2005). The carbon preference index (CPI) uses molecular ratios to infer the degradation status of organic sources in sediments. While high CPI values reflect the input of fresh terrestrial matter, thermally altered or extensively degraded OC approach a CPI of 1 (Bray & Evans, 1961; Collister et al., 1994; Freeman & Colarusso, 2001). In order to ensure comparability with other Arctic datasets (Bröder, Tesi, Andersson et al., 2016; Bröder, Tesi, Salvadó et al., 2016; Tesi et al., 2016; Vonk et al., 2017), we applied the CPI proxy defined by Bray and Evans (1961):

(1) $${\text{CPI}}_{25-33}=\frac{1}{2}*\left(\frac{\sum C_{25-33\;\text{odd}}}{\sum C_{24-32\;\text{even}}}+\frac{\sum C_{25-33\;\text{odd}}}{\sum C_{26-34\;\text{even}}}\right)$$

### 2.5 Data Analysis

All data analysis was performed using the Python programming language v.3.8. We applied the Spearman rank‐order correlation coefficient (*r*_S_) to account for the non‐normal character of sedimentological and geochemical parameters. Best‐fit linear and non‐linear regressions were identified using *R* ^2^, RMSE, and MAE (see Supporting Information S1). Due to unbalanced and small sample sizes, statistically significant differences between independent groups (surface vs. bottom) were tested using the non‐parametric, rank‐based Mann‐Whitney U Test. All statistical comparisons are reported at the 95% confidence interval (*p* < 0.05).

## 3 Results

### 3.1 Sedimentological Properties

Although silt constitutes the dominant particle phase in the Mackenzie River system, we observe notable contrasts in grain size distributions both spatially and as a function of water depth (Table S1). In general, fine sediments with unimodal grain size are found mainly in surface waters (<0.3 m), while the size of particles increases with depth and shifts toward a bimodal distribution. The coarsest sediments are present near the riverbed. The Mackenzie (*D*_84_: 120 ± 26 μm, *n* = 24; M ± SE) and the Peel Rivers (*D*_84_: 72 ± 24 μm, *n* = 12) supply primarily coarse sediments to the Mackenzie Delta, whereas the Arctic Red River is characterized by finer particle fractions (*D*_84_: 19 ± 3 μm, *n* = 15). The loss of larger particles due to gravitational settling and sorting during fluvial transfer results in a declined grain size distribution in the Mackenzie Delta (*D*_84_: 38 ± 6 μm, *n* = 30). In general, bank (*D*_84_: 122 ± 18 μm, *n* = 21) and bedload (*D*_84_: 227 ± 84 μm, *n* = 6) material consists of coarser grain sizes, largely composed of coarse silt and fine sand, and displays up to three modal sizes.

Measurements of SA are limited by the amount of sampling material and we focused on selected surface and deep suspended sediment samples (Table S1). The SA of suspended sediments in the northern Mackenzie River basin ranges from 4.8 to 31.7 m^2^ g^−1^, with an average of 18.6 ± 1.4 m^2^ g^−1^ (*n* = 31). Mineral SA is negatively correlated with grain size (*r*_S_ = −0.93, *n* = 31, *p* < 0.001) as has been reported in other large rivers (e.g., Bouchez et al., 2014). Surface area decreases with increasing water depth. We observe differences in SA between the sites. The Mackenzie River at the apex of the delta (11.8 ± 3.2 m^2^ g^−1^, *n* = 7) has the lowest SA values, while we find higher values in the Peel River (18.0 ± 2.7 m^2^ g^−1^, *n* = 5), Arctic Red River (24.8 ± 1.8 m^2^ g^−1^, *n* = 6), and the Mackenzie Delta (19.6 ± 2.0 m^2^ g^−1^, *n* = 13). Bank and bedload material display similar SA, averaging 9.7 ± 0.7 m^2^ g^−1^ (*n* = 21) and 9.6 ± 1.1 m^2^ g^−1^ (*n* = 6).

### 3.2 Bulk Organic Carbon and Nitrogen Contents

Particulate OC contents of the suspended load vary between 0.5 and 2.9 wt% and show no systematic trends with water depth (Table S1). We observe higher OC contents in the Arctic Red (2.1 ± 0.1 wt%, *n* = 18) and Peel Rivers (1.9 ± 0.1 wt%, *n* = 12) than in the Mackenzie River (1.0 ± 0.1 wt%, *n* = 23). The Mackenzie Delta is characterized by intermediate values of 1.5 ± 0.1 wt% (*n* = 29). Bank (1.0 ± 0.10 wt%, *n* = 24) and bedload (1.1 ± 0.3 wt%, *n* = 6) material contain on average less organic matter. Our findings are in good agreement with previously reported POC values from this system (Goñi et al., 2000, 2005; Guo et al., 2007; Hilton et al., 2015; Vonk et al., 2015).

Ratios of OC and SA (OC/SA) range from 0.5 to 2.2 mg m^−2^ for suspended sediments. In general, we note an increase in OC/SA values from surface to deeper river depth profile segments (Table S1). Averaged OC/SA are comparable for the Mackenzie (1.0 ± 0.2 mg m^−2^, *n* = 6), Arctic Red (0.9 ± 0.1 mg m^−2^, *n* = 6), Peel Rivers (1.2 ± 0.3 mg m^−2^; *n* = 5), and the Mackenzie Delta (1.0 ± 0.2 mg m^−2^, *n* = 13). The majority of the sediments are regarded as typical river suspended sediments (0.4–1.0 mg m^−2^; Blair & Aller, 2012). Samples exceeding this category (OC/SA > 1 mg m^−2^) are generally collected above the riverbed and are often characterized by high OC contents (OC > 1.5 wt%) or low SA (SA < 5.7 m^2^ g^−1^; CAN18_19–25 and CAN19_25). Bank (1.0 ± 0.1 mg m^−2^, *n* = 21) and bed sediment (1.1 ± 0.2 mg m^−2^, *n* = 6) OC/SA ratios are within the range of observed suspended sediments.

The N/OC values in the Mackenzie River system, ranging from 0.06 to 0.15 (0.10 ± 0.01, *n* = 82), decrease with increasing river depth (Table S1). The Arctic Red (0.09 ± 0.01, *n* = 18) and Peel Rivers (0.09 ± 0.01, *n* = 12) deliver materials with slightly lower N/OC ratios than the Mackenzie River (0.11 ± 0.01, *n* = 23) to the Mackenzie Delta (0.10 ± 0.01, *n* = 29). Bank (0.10 ± 0.01, *n* = 21) and bedload (0.09 ± 0.01, *n* = 6) resemble the suspended load signal.

### 3.3 Bulk Radiocarbon Compositions

**Figure 2:**
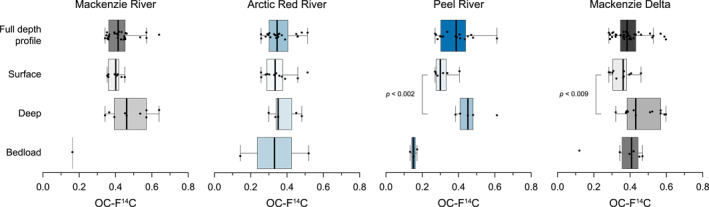
Boxplots displaying the bulk radiocarbon (OC‐F^14^C) composition of combined, surface, and deep suspended sediments as well as bedload material for sampling locations in the northern Mackenzie River basin. In addition, we utilize Mackenzie River depth profiles published by Hilton et al. (2015). Significant differences between surface and bottom samples are tested using the Mann‐Whitney U test. Boxes represent first and third quartiles, while whiskers display confidence intervals. Medians are shown as black lines.

Particulate OC associated with suspended sediment in the northern Mackenzie River basin generally exhibits depleted ^14^C contents, with F^14^C values ranging from 0.26 to 0.64 (0.40 ± 0.01, *n* = 82; Table S1). The Arctic Red (0.37 ± 0.02, *n* = 18) and Peel Rivers (0.39 ± 0.03, *n* = 12) transport more ^14^C‐depleted OC than the Mackenzie River at Tsiigehtchic (0.42 ± 0.02, *n* = 23) and in the delta (0.40 ± 0.02, *n* = 29). These values are consistent with previous work on this basin (Campeau et al., 2020; Goñi et al., 2005; Goñi, O’Connor et al., 2013; Guo et al., 2007; Hilton et al., 2015; Vonk et al., 2015). Mann‐Whitney U tests reveal significant differences in the observed OC‐F^14^C values of deep versus surface water column samples in the Peel River and the Mackenzie Delta: substantially aged OC is primarily present in surface waters, whereas more modern organic matter is transported above the riverbed (Figure 2 and Figures S1a–S1d in Supporting Information S1). More ^14^C‐depleted values are observed in bank (0.38 ± 0.03, *n* = 24) and bedload (0.31 ± 0.07, *n* = 8) material.

### 3.4 Biomarker Loadings

Solvent extractable *n*‐alkane loadings (ΣC_25−35_; biomarker abundance normalized to SA of a sample) range between 0.08 and 0.66 μg m^−2^ (0.29 ± 0.03 μg m^−2^, *n* = 31) in the suspended load (Table S1). We note the highest biomarker loadings in the Arctic Red (0.43 ± 0.08 μg m^−2^, *n* = 6) and Peel tributaries (0.37 ± 0.07 μg m^−2^, *n* = 5), whereas loadings in the Mackenzie River and Mackenzie Delta amount to 0.20 ± 0.05 μg m^−2^ (*n* = 7) and 0.23 ± 0.05 μg m^−2^ (*n* = 13), respectively. These values are comparable with *n*‐alkane loadings obtained from marine sediments on the East Siberian Shelf (Bröder, Tesi, Andersson et al., 2016; Bröder, Tesi, Salvadó et al., 2016; Tesi et al., 2016). *n*‐Alkane loadings appear to increase with increasing water depth. Carbon preference index values vary from 1.0 to 5.4. While average CPI values in the Mackenzie River, Peel River, and Mackenzie Delta may indicate higher contributions of fresh plant biomass (2.3–2.6), the Arctic Red River is characterized by more degraded (or a higher proportion of petrogenic) *n*‐alkane inputs (1.5 ± 0.1, *n* = 9).

Short‐chain *n*‐fatty acid loadings (ΣC_16–18_) display a minimum of 0.01 μg m^−2^ and a maximum of 0.99 μg m^−2^ (0.24 ± 0.05 μg m^−2^, *n* = 31). Average loadings in the Mackenzie River, the Peel River, and the Mackenzie Delta are comparable, ranging from 0.24 to 0.29 μg m^−2^, whereas loadings in the Arctic Red River are lower with values of 0.10 ± 0.03 μg m^−2^ (*n* = 6). Long‐chain *n*‐fatty acid loadings (ΣC_24–32_) vary between 0.06 and 1.41 μg m^−2^, with an average of 0.31 ± 0.06 μg m^−2^ (*n* = 31). Analogous to short‐chain *n*‐fatty acid loadings, long‐chain *n*‐fatty acid loadings in the Arctic Red River are lower (0.18 ± 0.06 μg m^−2^, *n* = 6). We detect higher loadings in the Mackenzie River (0.32 ± 0.12 μg m^−2^, *n* = 7), the Peel River (0.30 ± 0.10 μg m^−2^, *n* = 5), and the Mackenzie Delta (0.38 ± 0.13 μg m^−2^, *n* = 13). The highest *n*‐fatty acid loadings are usually observed close to the bedload.

## 4 Discussion

### 4.1 Evidence for a Plant Debris Rich Undercurrent

In the lower tributaries and the main delta channel of the northern Mackenzie River basin, we find that suspended sediments collected near the riverbed are characterized by elevated OC contents, higher OC/SA ratios, and are “younger” (OC‐F^14^C > 0.5) than finer‐grained sediment present in surface waters (Figure 2). Overall, this gives rise to a marked inversion of the ^14^C activity and OC/SA ratios with depth in the river. Beneath the suspended sediment load, the bedload is often characterized by strongly aged OC and low OC/SA ratios. The “younging” of suspended load OC toward the riverbed appears to be a persistent feature of the northern Mackenzie River system and has been noted during several periods of annual flooding (Hilton et al., 2015). These observations contrast with generally higher mean OC‐F^14^C values that are more or less invariant with increasing water depth in the Amazon (Bouchez et al., 2014) and the Danube Rivers (Freymond, Lupker et al., 2018) (Figures S1e and S1f in Supporting Information S1). This vertical trend is the opposite of that found in tributaries of the Ganges‐Brahmaputra watershed, where OC‐F^14^C signatures decrease with increasing water depth (Galy, Beyssac et al., 2008) (Figure S1g in Supporting Information S1). To understand these patterns and their implications for the transport and fate of POC in large rivers, we must first assess the sources of organic matter.

**Figure 3:**
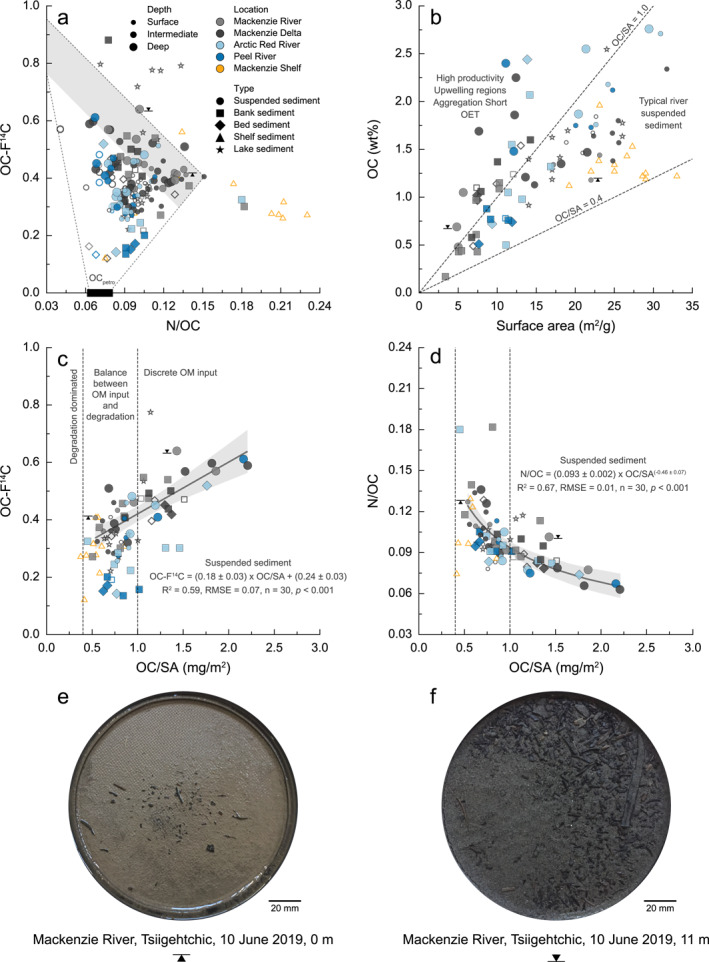
Relationships between bulk organic carbon (OC), nitrogen (N), and surface area (SA), with (a) radiocarbon (OC‐F^14^C) against N/OC, (b) OC (wt%) against SA (m^2^ g^−1^), (c) OC‐F^14^C, and (d) N/OC ratio against OC/SA (mg m^−2^). Samples are color‐coded for their respective sample location. Closed symbols represent this study, while open symbols denote literature values. Circles display suspended, squares bank, and diamonds bed sediments (Hilton et al., 2015). Shelf sediments are depicted as yellow triangles (Drenzek et al., 2007; Goñi et al., 2005; Goñi, O’Connor et al., 2013; Hilton et al., 2015; Vonk et al., 2015), lake sediments as stars (Vonk et al., 2015, 2016). In addition, suspended sediments are scaled to surface, intermediate, and deep river depth segments. The dashed lines in (a) indicate the compositions expected by mixing rock‐derived petrogenic (black rectangle; Hilton et al., 2015) and biospheric OC (gray shading). The shading depicts the trend from a peat core in western Canada (Kuhry & Vitt, 1996). Depositional regimes are defined by Blair and Aller (2012). Data are fit with linear (c) and power law (d) regressions. Suspended sediment retrieved from Mackenzie River (e) surface and (f) bottom waters were collected on PES filters (diameter = 142 mm, pore size = 0.22 μm) in 2019. Black triangles denote these surface and bottom samples.

In the Mackenzie River, we observe an inverse relationship between the N/OC ratio and OC‐F^14^C values, as initially noted by Hilton et al. (2015) (Figure 3a). Low relative N abundance is typical of “fresher” polysaccharide‐ and lignin‐rich terrestrial organic matter (Bröder et al., 2021; Goñi et al., 2000), while high relative N contents are often linked to the degradation of soil organic matter and enrichment in microbial residues (Kuhry & Vitt, 1996; Schädel et al., 2014; Strauss et al., 2015). In comparison to other climatic zones, Arctic soil OC experiences longer residence times (Frank et al., 2012). The preservation of soil organic matter is driven by organo‐mineral interactions (e.g., Hemingway et al., 2019; Lützow et al., 2006; Prater et al., 2020), waterlogging (Oades, 1988), and sub‐zero temperatures (Kaiser et al., 2007; Ping et al., 2015) that all slow microbial degradation and promote aging of soil OC. Deep soil organic matter is mobilized by river bank incision (Carson et al., 1998), slumping, and landsliding induced by permafrost degradation (Kokelj et al., 2017). Given the fine‐grained character of the OC, the higher N/OC, and lower OC‐F^14^C values, we infer that sediments carried in surface waters are likely sourced from deeper/older soils. Previous studies demonstrated that the majority of OC carried by the suspended load of Mackenzie River and Delta are derived from aged biospheric inputs (Hilton et al., 2015; Vonk et al., 2015, 2019), while the Arctic Red and Peel Rivers receive relatively higher inputs of rock‐derived (petrogenic) OC (Millot et al., 2003; Yunker et al., 2002). The presence of aged organic matter in these river systems likely represents a mixture of petrogenic OC and more modern sources (plants, algae; Goñi et al., 2005; Hilton et al., 2015; Vonk et al., 2015, 2016, 2019). Within deeper parts of the water column, suspended sediments tend to be enriched in OC, corresponding to the presence of more recent (but still centuries old), less extensively degraded (lower N/OC) plant‐derived biomass (Figure 3a).

These inferences on OC sources are supported by the OC/SA ratios of the materials (Freymond, Kündig et al., 2018; Wakeham & Canuel, 2016). Organic matter OC/SA ratios and OC‐F^14^C of suspended sediments show a positive relationship (*r*_S_ = 0.59, *n* = 30, *p* < 0.001; Figure 3c). While the river surface sediments appear to consist of fine‐grained (high SA; Figure 3b), aged material, suspended sediments from deeper transects display notable increases in OC/SA. This is not an increase in “carbon loading,” but instead reflects an increase in the proportion of discrete organic matter‐derived particles (Figures 3b–3d). Macroscopic observations made during sample collection and filtration further support this geochemical evidence: surface water suspended sediments primarily consisted of fine‐grained particles and a low abundance of visible, discrete plant fragments (Figure 3e), while considerable accumulations of visible wood and plant debris in a silt‐sandy matrix were retrieved near the bed of the Mackenzie River within the same depth profile (Figure 3f).

In contrast to the above observations, suspended sediments collected from depth profiles from the Amazon and Danube Rivers appear to be better mixed and consist primarily of modern OC derived from recent soil and plant biomass. The overall modern OC‐^14^C signature complicates the source allocation of vegetation debris and organic matter associated with mineral matrices in those rivers. Rivers traversing the Himalayan range transport significant amounts of petrogenic OC, while biospheric organic matter is subjected to protracted storage and aging (French et al., 2018; Galy & Eglinton, 2011). Consequently, modern, discrete fragments of POC buried in the Bengal fan are mainly sourced from the lowlands (French et al., 2018; Lee et al., 2019).

**Figure 4:**
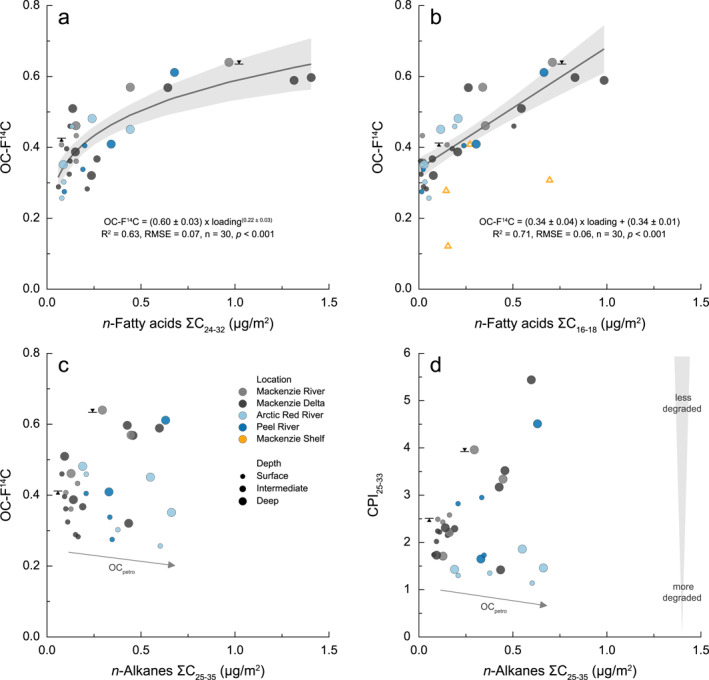
Suspended sediment (a) long‐chain and (b) short‐chain *n*‐fatty acid loadings (μg m^−2^) displayed as a function of radiocarbon (OC‐F^14^C). Data are fit with power law and linear regressions. *n*‐Alkane loadings (μg m^−2^) plotted against (c) OC‐F^14^C and (d) carbon preference index (CPI). Suspended sediments are color‐coded for locations and are scaled to river depth. Shelf sediments are from Goñi et al. (2005). Black triangles indicate the surface and bottom suspended sediments displayed in Figures 3e and 3f.

The occurrence of wood‐laden currents has previously been described in the Madre de Dios River where particulate lignin accumulated in the lower water column (Feng et al., 2016; Repasch et al., 2022). However, we find that the transport of coarse organic matter in the Mackenzie River basin is not limited to woody (lignin‐rich) debris. Biomarker loadings of long‐chain *n*‐fatty acids (ΣC_24−32_) derived from cuticular leaf waxes are elevated in suspended sediments collected from deep river profiles, indicating the accumulation of significant amounts of non‐woody plant tissue above the riverbed (Figure 4a). These findings are consistent with previous studies on temperate rivers where high biomarker yields were associated with coarser, less‐degraded plant biomass (Goñi, Hatten et al., 2013; Hatten et al., 2012). The addition of this organic matter source can explain the increase in OC‐F^14^C values for suspended sediment near the riverbed (Figures 2 and 3).

While long‐chain *n*‐fatty acids are typically produced by vascular plants, short‐chain *n*‐fatty acids are non‐specific and may be derived from higher plant, bacterial, or algal activity (Drenzek et al., 2007; Feng, Benitez‐Nelson et al., 2013; Volkman et al., 1998). Irrespective of their source, they are typically regarded as labile organic compounds with faster degradation rates (Canuel & Martens, 1996; Tao et al., 2016). Elevated loadings of short‐chain lipids in deep river depth transects further aid the hypothesis that OC is sourced from surface soil layers (Figure 4b) (Feng et al., 2015; Feng, Vonk et al., 2013).

In addition to aged soil organic matter, as inferred from the N/OC values (Figure 3a), another source of ^14^C‐depleted OC is known to be important in the Mackenzie River basin: petrogenic or rock‐derived POC (Hilton et al., 2015; Horan et al., 2019; Yunker et al., 1993, 2002). Input of ^14^C‐free, petrogenic OC can explain the ^14^C‐depletion in the bedload samples (Galy, Beyssac et al., 2008), but may also be more widely distributed in the suspended load as observed in other systems (Galy et al., 2015 and references therein). The loadings of *n*‐alkanes are sensitive to contributions of bedrock‐derived (thermally mature) hydrocarbons (Figure 4c) (Tao et al., 2015). In particular, the Arctic Red and Peel River watersheds contain successions of marine shale abundant in fossil OC (Millot et al., 2003). On the Mackenzie Shelf, Drenzek et al. (2007) found that ^14^C ages of *n*‐alkanes extracted from surface sediments are substantially older than those of long‐chain *n*‐fatty acids suggesting a petrogenic origin. A cross‐plot of *n*‐alkane loadings and corresponding CPI values (Figure 4d) confirms the input of petrogenic OC, as we observe two diverging trends. Suspended sediments collected from the Mackenzie River and Delta display rather high CPI values which increase with *n*‐alkane loadings. In contrast, the increase in loadings accompanied by a decrease in CPI values observed in the Arctic Red River likely reflects substantial contributions of petrogenic OC or the input of highly degraded biospheric organic matter.

Given our sampling setup, we are not able to determine the spatial and temporal extent of this sediment‐ and plant debris‐laden undercurrent. However, the accumulation of modern OC collected from various depth profiles within the Mackenzie River mainstem and its tributaries as well as the annual reoccurrence of this feature suggest a widespread and systematic transport of submerged plant debris near the river channel bottom. Here, we now consider the mechanisms behind this phenomenon and its implications for fluxes and fates of organic matter.

### 4.2 Entrainment and Transport of Coarse Particulate Organic Matter

A range of geomorphic processes mobilize and deliver litter and wood fragments to fluvial systems, including hillslope failure, bank erosion, flooding, and storm‐driven surface runoff (Hilton et al., 2011; Hilton, Galy, Hovius et al., 2008; Ruiz‐Villanueva et al., 2014; Turowski et al., 2016; West et al., 2011). While the recruitment and transport of large woody debris (>10 cm; driftwood) are increasingly well documented (Kramer et al., 2017; Ruiz‐Villanueva et al., 2019; Seo et al., 2008; West et al., 2011; Wohl, 2017), the riverine transfer of submerged plant‐rich debris is less constrained (Turowski et al., 2013, 2016). In the Mackenzie River system, the mobilization of coarse organic matter is largely driven by the spring freshet (Carson et al., 1998; Hill et al., 2001). Flooding induced by snowmelt and the ice‐breakup erodes and flushes significant amounts of woody and non‐woody plant fragments into adjacent channels. In high‐latitude environments, thaw‐driven destabilization of soils and sediments increasingly shapes Arctic landscapes (Nitze et al., 2018). These thermo‐erosional mass wasting events, including gullies, active layer detachment slides, and retrogressive thaw slumps actively couple hillslopes with aquatic systems, entraining a wide range of vegetation and permafrost‐derived POC (Bröder et al., 2021; Kokelj et al., 2021; Shakil et al., 2020).

Most fresh plant material has a bulk density less than water and will initially float. However, waterlogging will induce sinking and can occur either by prolonged in‐stream transport and storage or by saturated conditions in the landscape (Nichols et al., 2000; Turowski et al., 2013, 2016). While we cannot rule out within‐channel waterlogging of particulate OC, the ^14^C content of the plant‐debris enriched samples is ∼0.57–0.64 (Figure 4), corresponding to conventional ^14^C ages of ∼3500–4500 years. This suggests this bulk material has spent a significant time in the catchment prior to arrival at the delta head. Aging could occur if material is deposited within river channel bars, but perhaps more likely occurs on the landscape during soil‐formation processes.

**Figure 5:**
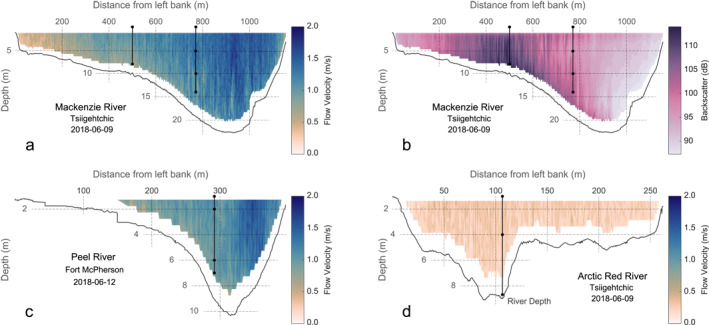
Cross‐channel profiles acquired using Acoustic Doppler Current Profiler of downstream (a) flow velocities and (b) backscatter for the Mackenzie River, and flow velocities for the (c) Peel and (d) Arctic Red Rivers. River depth profiles are represented as black lines, while sample depths correspond to black dots. Note differences in channel depths and widths.

Regardless of the mechanism and location of waterlogging, submerged debris is subjected to sorting, separation, and breakdown during transport within the river. Waterlogged vascular plant debris has a similar hydraulic behavior to silt or sand and is often concentrated in the coarse‐grained lithogenic fraction (Bianchi et al., 2002, 2007; Leithold & Hope, 1999; Wakeham et al., 2009). Flume experiments showed that despite the increased density, waterlogged fragments often remain in suspension with sufficient flow velocity (>0.6 m s^−1^, Figure 5) (Nichols et al., 2000) supported by the planar and elongated nature of these particles (Braudrick et al., 1997; Nichols et al., 2000) and tend to accumulate above the riverbed.

While particle distributions in river profiles characterized by maximum flow velocities appear relatively homogenous, we observe an accumulation of sediments and grain size separation toward the inner bend induced by abating flow strengths and gravitational settling (Figure 5a). This area of elevated particle concentration is reflected by high ADCP backscatter values in the Mackenzie River (Figure 5b). In addition to the primary factor of flow strength controlling particle flotation and settling in the water column, we argue that the formation of a “vegetal” undercurrent on the inner bend is likely supported by secondary flow motions. In meandering river systems, secondary or helical flow can divert coarse particles toward the inner bank forming areas of dense, sediment‐laden plumes (Azpiroz‐Zabala et al., 2017; Sumner et al., 2014). When passing through a bend, curvature‐induced centrifugal acceleration drives surface waters outwards (superelevation) producing pressure gradients near the riverbed which in turn forces the flow toward the inner bend (Thorne et al., 1985). The resulting helical circulation strongly impacts particle transport and ultimately the morphology of the channel (Figure 5). A sufficient centrifugally driven pressure gradient invoked, for example, by flooding, causes sustained overturning and mixing of suspended sediment forming an undercurrent rich in coarse lithogenic particles (Azpiroz‐Zabala et al., 2017). Given the similarities in the hydrodynamic behavior of vascular plant debris and coarse sediment, these event‐driven processes may pose as a pathway for rapid and efficient plant biomass transport that augments the export of older, more degraded mineral‐hosted biospheric carbon associated with fine‐grained suspended sediments.

Both sedimentological and geochemical evidence suggests the existence of undercurrents rich in plant debris in the Mackenzie Delta and its largest sediment suppliers—the Mackenzie and Peel Rivers (Figures 5a–5c). However, we note that the Arctic Red River does not show any systematic particle separation, and suspended sediments in the Arctic Red River appear to be rather homogenous and well‐mixed during the spring freshet (Figures 2 and 5d). We suggest that this different behavior does not reflect a variation in potential OC sources in the Arctic Red River, but instead that it relates to the morphology and hydrodynamic characteristics of the channel (Figure 5d). The Arctic Red River at Tsiigehtchic has a relatively shallow (max depth 5–9 m) and low slope river channel, and thus lacks sufficient flow velocity to support the entrainment and transport of coarser, discrete particles in the water column. Coarse‐grained sediments may be deposited upstream within the sedimentary system, and this warrants further research.

### 4.3 Fate of Plant‐Derived Organic Carbon Exported by the Mackenzie River

The physical and chemical composition of surficial sediments from cores retrieved on the Beaufort Shelf differ markedly from those of the Mackenzie River suspended sediment load (Figure 3) (Goñi et al., 2005; Goñi, O’Connor et al., 2013; Hilton et al., 2015; Vonk et al., 2015). While these shelf sediments are enriched in N due to the contribution of marine primary productivity, their OC signature is generally more degraded and depleted in ^14^C, suggesting protracted storage on the continental margin. In comparison to fluvial suspended sediments, shelf sediments contain higher proportions of clay (Goñi et al., 2005; Vonk et al., 2015) and are devoid of discrete terrestrial organic matter, as noted in sediment cores retrieved in front of the Mackenzie Delta (Hilton et al., 2015; O’Regan et al., 2018; Richerol et al., 2008). The lack of litter and wood debris on the Mackenzie Shelf raises questions regarding the depositional regime and fate of coarse OC.

Given the paucity of collected marine sediments from the Mackenzie Shelf, sampling strategies may have missed potential depositional locations favoring the settling of discrete organic matter (Figure 1). However, studies from the Amazon (S. Sun et al., 2017), Eel (Leithold & Hope, 1999), Mississippi (Bianchi et al., 2002; Goñi et al., 1997), and Yangtze River (X. Sun et al., 2021) margins show that coarse, vascular plant debris is largely thought to be retained on the inner shelf proximal to the outlet, while fine‐grained, mineral‐bound OC may be preferentially advected across the shelf (Bröder et al., 2018; Goñi et al., 2005; Tesi et al., 2016). On the Mackenzie Shelf, lignin and vascular plant biomarkers further illustrate a cross‐shelf decrease in woody and non‐woody plant tissue with increasing distance from the delta (Belicka et al., 2004; Drenzek et al., 2007; Goñi et al., 2005, 2000; Goñi, O’Connor et al., 2013; Macdonald et al., 1998). Similar observations have been made on the Siberian Shelves where coarse OC is primarily deposited in shallow waters (Bröder et al., 2018; Bröder, Tesi, Salvadó et al., 2016; Dickens et al., 2011; Sparkes et al., 2016; Tesi et al., 2014, 2016; Vonk et al., 2010).

Recent studies recognize the significance of submarine canyons for the export of coarse terrestrial‐derived OC and its burial in submarine fans (Hage et al., 2020; Kao et al., 2014; Lee et al., 2019; Sparkes et al., 2015). Canyons may potentially serve as active bypass structures that “circumvent” the continental shelf and funnel sediments and organic matter directly to marine basins (Canals et al., 2006; Paull et al., 2018; Zheng et al., 2017). The Mackenzie Trough is the only submarine canyon in the Arctic Ocean that links a river outlet to the shelf slope and thus may represent an important conduit piping sediment offshore (Harris & Whiteway, 2011). However, minimal sediment accumulation rates in the Canada Basin indicate limited export of terrestrial material to the abyssal basin (Backman et al., 2004; Griffith et al., 2012; Hwang et al., 2015).

The preferential export of fine‐grained lithogenic particles and OC combined with the limited burial of discrete plant biomass in pelagic sediments suggest that the majority of coarse organic matter is likely retained within the subaerial and subaqueous delta plain. Changes in the flow regime along the land to ocean aquatic continuum induces hydrodynamic sorting and distinct deposition patterns (Bao, Uchida et al., 2018; Bao, Zhao et al., 2019; Bianchi et al., 2002; Emmerton et al., 2008; Keil et al., 1994; Wakeham et al., 2009). The shallowing of channels toward the Outer Delta (Emmerton et al., 2008) decelerates flow, triggering the settling of coarse organic matter into the bedload. The Mackenzie Delta consists of a multitude of shallow lakes and channels which capture and store vast amounts of water and sediment (Carson et al., 1999; Emmerton et al., 2007; Hill et al., 2001). Although lakes may be considered as potential depocenters for detrital plant fragments (Vonk et al., 2015, 2016, 2019), reduced hydraulic connectivity and hydrodynamic sorting within the river water column promote the preferential inflow of surface waters enriched in fine, ^14^C‐depleted OC (Section 4.1, Figure 3), while sills prevent coarser, more modern organic matter transported near the riverbed from entering low and high‐closure lakes.

Historical sedimentary records provide further insights and also demonstrate the significance of plant debris transport and deposition in alluvial environments under different climate regimes. The Pliocene Beaufort Formation located in the Canadian High Arctic contains cross‐bed lenses composed of sand and abundant fine woody debris (<2.5 cm; Davies et al., 2014). These lenses correspond to typical river scour and bar facies. The warm climatic interval enabled the northward migration of boreal forests, enhancing the supply and accumulation of detrital wood in floodplains (Davies et al., 2014; O’Regan et al., 2018). The increased burial of undegraded plant debris under warm Pliocene conditions implies that alluvial settings can preserve substantial amounts of OC. Furthermore, the Beaufort Formation may potentially serve as an analog for a warming Arctic.

Degradation processes in the Mackenzie River floodplain and delta may likely remove plant biomass driven by microbial and fungal decomposition (Dao et al., 2022; Scheingross et al., 2021). However, the efficacy of microbial metabolism is markedly subdued by persistent winter darkness, humidity, and low temperatures promoting the accumulation and water saturation of plant detritus in Arctic watersheds. Although stability and turnover times of plant‐derived biomarkers are associated with high uncertainties (Jex et al., 2014; Schmidt et al., 2011), lignin phenols and plant lipids are often preserved in sedimentary and soil sequences indicating an enhanced resistance to remineralization (e.g., T. I. Eglinton & Eglinton, 2008). Based on our proposed transport mechanism, submerged, water‐saturated discrete fragments of plant debris are carried within an undercurrent above the riverbed, effectively decoupled from aerial degradation processes. The rapid mobilization, transfer, and deposition of OC during the freshet further reduce storage and oxygen exposure times. Co‐transport and deposition with substantial amounts of suspended sediment ensure high burial efficiency as the Mackenzie River discharges the annual sediment load as a single event within weeks after the ice break‐up (Carson et al., 1999; Holmes, 2002; Macdonald et al., 1998). The significance of sediment shunting in response to episodic high discharge events has been recognized in small mountainous river systems (Milliman & Syvitski, 1992), where steep basin morphology and high erosion rates promote the export of coarse organic matter and its subsequent burial on continental margins (Hilton, Galy, & Hovius, 2008; Leithold & Hope, 1999; Seo et al., 2008; Sparkes et al., 2015; West et al., 2011). We argue that the swift export and high sediment accumulation rates facilitate the burial of biospheric OC within the Mackenzie Delta, reduce oxidation, and promote the preservation and effective draw‐down of atmospheric CO_2_ (R. A. Berner, 1982; Galy et al., 2007, 2015; Hedges & Keil, 1995).

### 4.4 Implications for Regional Carbon Budgets

**Figure 6:**
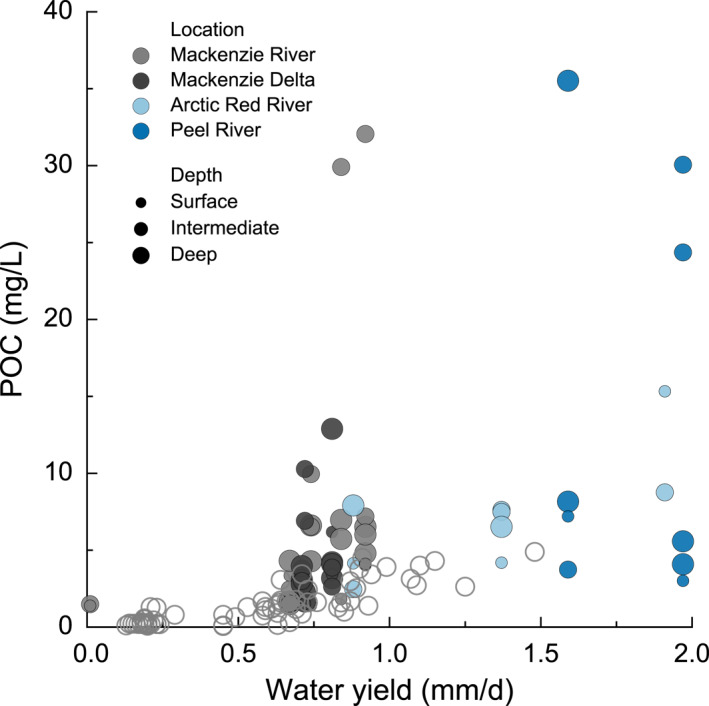
Particulate organic carbon concentrations (mg L^−1^) plotted as function of water yield (mm d^−1^). Suspended sediments are color‐coded for locations and are scaled to river depth. Open circles represent the PARTNERS and ArcticGRO data set at Tsiigehtchic from 2004 to 2019 (Holmes et al., 2021).

This study reveals that mineral‐hosted and discrete biospheric OC in the riverine suspended load is subject to hydrodynamic sorting processes that result in diverging transport pathways and depositional fates. Estimations of sediment discharge and OC fluxes commonly rely on suspended sediment samples collected from river surface waters (Milliman & Farnsworth, 2013). This sampling strategy neglects vertical variations in suspended sediment content and composition, and in the case of the Mackenzie River, it fails to account for the accumulation of coarse organic matter in deeper river segments, resulting in an underestimation of OC export. Lateral gradients in suspended sediment discharge may be equally significant. In large rivers, particles are further influenced by secondary flows (e.g., helical flow) adding to the complexity of sediment transport patterns (Baronas et al., 2020). Consequently, a single river depth profile consisting of evenly spaced depth samples may be insufficient to accurately resolve suspended sediment and associated geochemical fluxes. Figure 6 demonstrates that statistical means obtained from measurement of depth‐integrated samples retrieved from the Mackenzie River at Tsiigehtchic by the Pan‐Arctic River Transport of Nutrients, Organic Matter, and Suspended Sediments (PARTNERS) and the Arctic Great Rivers Observatory (ArcticGRO) programs (Holmes et al., 2021; McClelland et al., 2016) appear not to capture sediment‐laden plumes. Specifically, POC in turbid transects collected from 2017 to 2019 in this study often display much higher concentrations for a given water yield than samples from the ArcticGRO data set. We measure similar concentrations of POC (mg L^−1^) in surface samples as those reported by McClelland et al. (2016), yet find concentrations at depth that surpass those in the surface by a factor of 10. Although it is beyond the scope of the current study, a combination of strategically selected depth transects and modeling would be required to accurately quantify OC compositions and fluxes (Baronas et al., 2020).

The permanent burial of biospheric OC in deltaic and marine sediments constitutes a sink of atmospheric CO_2_ and thereby contributes to long‐term mitigation of climate change (R. A. Berner, 1982; Burdige, 2005; Schlünz & Schneider, 2000; Smith et al., 2015). Current carbon budgets primarily focus on OC accumulating in fine‐grained sedimentary deposits and disregard coarser‐grain depositional environments due to the notion that their high permeability and greater oxygen penetration leads to efficient organic matter remineralization (Burdige, 2005, 2007). However, recent studies have shown that under appropriate depositional conditions, coarse‐grained matrices are indeed capable of actively preserving coarse plant biomass in marine sediments on geological timescales (Davies et al., 2014; Hage et al., 2020; Lee et al., 2019; Sparkes et al., 2015). Similar, coarse matrices of sand bars and riverbeds may equally preserve waterlogged litter and wood in deltaic environments (Nichols et al., 2000), thereby representing an underestimated sink for biospheric OC.

With continued climate warming, OC sources and sinks are subjected to alterations in their extent and efficiency (AMAP, 2017; IPCC, 2021; Jorgenson et al., 2006; Post et al., 2019; Schuur et al., 2015) likely altering the supply, export, and burial of plant‐derived debris. Increasing air and soil temperatures, soil moisture, and the lengthening of growing seasons favor the northward migration of shrub and tree populations resulting in a widespread Arctic greening (e.g., Arndt et al., 2019; L. T. Berner et al., 2020; Ju & Masek, 2016; Myers‐Smith et al., 2020; Myneni et al., 1997). On the other hand, elevated temperature conditions may promote the accelerated decay of plant detritus prior to its transfer to the aquatic system. While increased precipitation will further the efficacy of surface runoff (Anderson et al., 2018; Bintanja et al., 2020), the loss of permafrost will both enhance physical erosion (e.g., Bröder et al., 2021) and hydrological connectivity in soils, resulting in heightened discharges of water, nutrients (Doxaran et al., 2015; Drake et al., 2018; McClelland et al., 2016; Rood et al., 2017; Tank et al., 2016), and sediments to the Arctic Ocean (Kokelj et al., 2021; Wild et al., 2019). In deltaic settings, permafrost thaw and changes in ice thickness will fundamentally affect Arctic delta morphology, the dispersal, and retention of flow and sediment. Numerical modeling experiments show that ice and permafrost loss will allow the lateral migration of channels and delta propagation, but simultaneously decrease overbank flooding and offshore transport (Lauzon et al., 2019; Piliouras et al., 2021). These results suggest an expansion of the depositional environment for plant debris in the Outer Delta. In response to impending warming, observations and predictions point toward an increase in both sources and sinks for coarse organic matter and stress the significance of non‐woody and woody plant tissue on regional carbon budgets.

## 5 Conclusions

We investigated spatial and water depth‐related variations in physical properties, bulk, and molecular organic geochemical characteristics of suspended sediments from the Mackenzie River system. Macroscopic observations, enriched OC‐F^14^C compositions, and high bulk OC and biomarker loadings suggest the near‐bed accumulation of coarse fragments of relatively fresh vascular plant‐derived debris. Light and climatic conditions in polar regions minimize microbial decomposition rates resulting in the aggregation of plant detritus and favor waterlogging within the watershed. We speculate that the spring freshet mobilizes and entrains coarse plant biomass into the river where sufficient flow strength and buoyant properties keep submerged debris in suspension close to the riverbed. Turbulences, such as helical flow motion, resulting from competing forces when passing through a river bend, support conditions that allow the maintaining of coarse lithogenic and organic particles in suspension, creating sediment‐laden undercurrents. The absence of coarse organic matter in Mackenzie Shelf sediments, as well as in offshore hemipelagic deposits, suggests that these materials drop out of suspension under declining flow regimes and are deposited in hydrodynamically quiescent regions of the subaerial and subaqueous delta. The sequestration of discrete fragments of biospheric OC in the Mackenzie Delta is promoted by the rapid export and high sediment accumulation rates associated with the spring freshet.

The widespread and reoccurring nature of a fluvial undercurrent enriched in coarse, relatively fresh plant debris sheds light on a poorly understood yet potentially important component of the terrestrial carbon cycle. The underlying processes are obscured in current river sampling strategies that largely focus on the surface suspended load, leading to incomplete capture of depth and lateral variations in sediment and OC transport, and large uncertainties in associated budgets. It remains to be determined whether such vegetal undercurrents are limited to specific fluvial systems and hydrodynamic conditions. Analysis of OC compositions and concentrations across multiple depth profiles and across‐river transects in different river systems is required to accurately estimate the importance of these processes with respect to the global transfer of biospheric POC from land to ocean.

## Conflict of Interest

The authors declare no conflicts of interest relevant to this study.

## Supporting information

Supporting Information S1

Table S1

## Data Availability

All data generated from 2013 to 2019 are openly available in the EarthChem Library via https://doi.org/10.26022/IEDA/112218.
