# Supplementary material for: Vegetal Undercurrents—Obscured Riverine Dynamics of Plant Debris
Source: J Geophys Res Biogeosci. 2022 Mar 28;127(3):e2021JG006726. doi: 10.1029/2021JG006726 (PMC9285624; doi:10.1029/2021JG006726)
Supplement: Supplementary file 1 — Supporting Information S1 [file JGRG-127-0-s001.pdf]

AGU PUBLICATIONS

Journal of Geophysical Research: Biogeosciences

Supporting Information for

Vegetal Undercurrents – Obscured Riverine Dynamics of Plant Debris

Melissa S. Schwab[1,2], Robert G. Hilton[3], Negar Haghipour[1,4], J. Jotautas Baronas[5] and Timothy I. Eglinton[1]

[1]Department of Earth Sciences, ETH Zurich, Sonneggstrasse 5, 8092 Zurich, Switzerland

[2]Now at Jet Propulsion Laboratory, California Institute of Technology, 4800 Oak Grove Drive, Pasadena, CA 91109, USA

[3]Department of Earth Sciences, University of Oxford, South Parks Road, Oxford OX1 3AN, UK

[4]Laboratory of Ion Beam Physics, ETH Zurich, Otto-Stern-Weg 5, 8093 Zurich, Switzerland

[5]Institut de Physique du Globe de Paris, Université de Paris, 1 rue Jussieu, 75005 Paris, France

Contents of this file

Text S1

Figure S1

Tables S1 to S3

S1. Regression analysis

We applied linear, quadratic, and non-linear fitting functions including exponential, power law, logarithmic, and Michaelis-Menten equations to assign 'best fit' models to our dataset. The performance of each regression model was evaluated based on the coefficient of determination (R^2), the root mean squared error (RMSE), and the mean absolute error (MAE). While the R^2 indicates the precision of the standard regression type, RMSE and MAE represent the accuracy. These goodness-of-fit tests suggest the relationships between the organic carbon and surface ratio (OC/SA), short-chain (ΣC_{16-18}) and long-chain (ΣC_{24-32}) *n*-fatty acid loadings, and OC-$F^{14}C$ are foremost represented by a quadratic fit (**Table S3**). However, the extrapolation of a quadratic function results in a decrease of OC-$F^{14}C$ values with increasing OC/SA or *n*-fatty acid loadings. This assumption is not supported by evidence and misrepresentative of this natural system. We therefore favor power and linear function to describe the relationship between predictors and response variables.

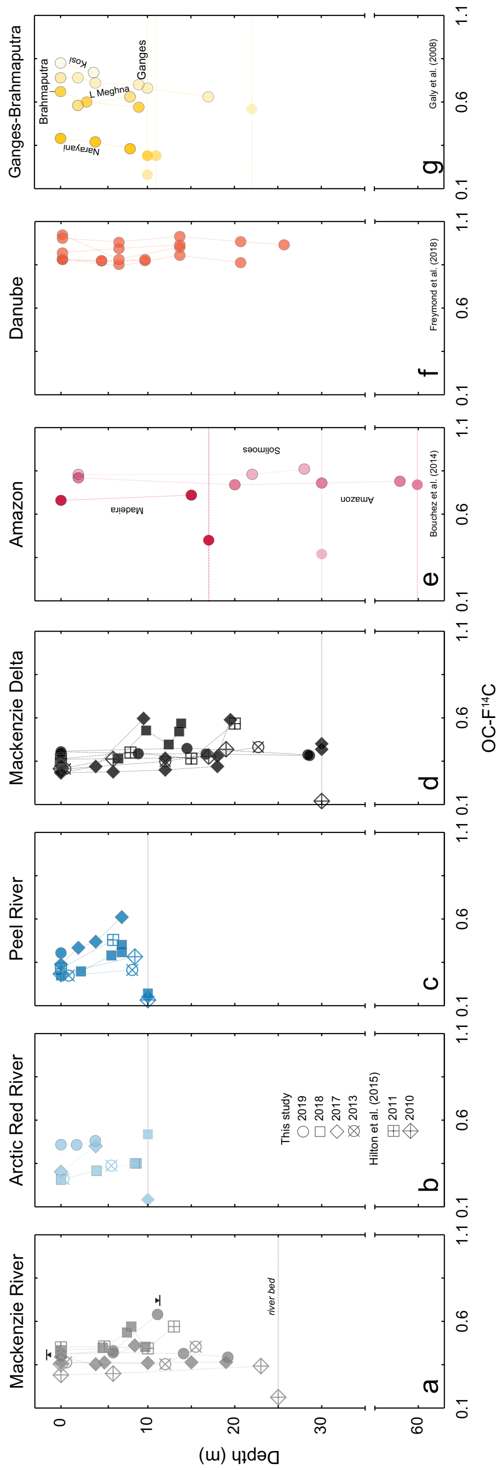

Figure S1. Radiocarbon activity (OC-F^{14}C) of suspended sediment and bedload material in depth profiles. Depth profiles are shown for different sampling years for (**a**) the Mackenzie River at the apex of the delta, the (**b**) Arctic Red and (**c**) Peel Rivers, and (**d**) the Mackenzie River in the delta. Depth profiles of (**e**) the Amazon (Bouchez et al., 2014), (**f**) the Danube (Freymond et al., 2018), and (**g**) the Ganges-Brahmaputra system (Galy, Beyssac, et al., 2008) are depicted to allow direct comparisons.

Table S1. Sedimentological properties, bulk N and C elemental and isotopic compositions, *n*-alkane ($\Sigma C_{25\text{-}35}$), short-chain ($\Sigma C_{16\text{-}18}$), and long-chain ($\Sigma C_{24\text{-}32}$) *n*-fatty acid loadings as well as *n*-alkane carbon preference index (CPI) for suspended, bank, and bedload material.

Table S2. Nonparametric Mann-Whitney U Test comparing the radiocarbon content (OC-$F^{14}C$) of surface and bottom suspended sediment samples for different locations.

	Mann-Whitney U Test		
	n	W	*p*-value
Mackenzie River<br>Tsiigehtchic	18	54	0.093
Arctic Red River<br>Tsiigehtchic	18	45	0.437
Peel River<br>Fort McPherson	11	29	**0.009**
Mackenzie Delta<br>Middle Channel	24	122	**0.002**

Table S3. Statistical performance of linear and non-linear regressions. Dark blue lines indicate 'best fit' functions, while light blue lines illustrate 'best fit' functions with informative priors.

Model	R^2	RMSE	MAE
**OC/SA vs. OC-$F^{14}C$**			
Linear	0.587	0.069	0.056
Quadratic	**0.589**	**0.069**	**0.056**
Exponential	0.577	0.070	0.057
Power law	0.581	0.070	0.056
Logarithmic	0.562	0.071	0.057
Michaelis-Menten	0.566	0.071	0.057
**OC/SA vs. N/OC**			
Linear	0.603	0.012	0.010
Quadratic	0.660	0.011	0.009
Exponential	0.632	0.012	0.010
Power law	**0.674**	**0.011**	**0.009**
Logarithmic	0.661	0.011	0.009
Michaelis-Menten	0.639	0.012	0.010
***n*-fatty acids (ΣC_{24-32}) vs. OC-$F^{14}C$**			
Linear	0.563	0.071	0.062
Quadratic	**0.653**	**0.063**	**0.052**
Exponential	0.525	0.074	0.064
Power law	0.630	0.065	0.056
Logarithmic	0.626	0.066	0.056
Michaelis-Menten	0.572	0.071	0.059
***n*-fatty acids (ΣC_{16-18}) vs. OC-$F^{14}C$**			
Linear	0.710	0.058	0.046
Quadratic	**0.762**	**0.053**	**0.044**
Exponential	0.668	0.062	0.048
Power law	0.703	0.059	0.050
Logarithmic	0.657	0.063	0.054
Michaelis-Menten	0.425	0.082	0.068

References

Bouchez, J., Galy, V., Hilton, R. G., Gaillardet, J. Ô., Moreira-Turcq, P., Pérez, M. A., et al. (2014). Source, transport and fluxes of Amazon River particulate organic carbon: Insights from river sediment depth-profiles. *Geochimica et Cosmochimica Acta*, *133*, 280–298. https://doi.org/10.1016/j.gca.2014.02.032

Freymond, C. V., Lupker, M., Peterse, F., Haghipour, N., Wacker, L., Filip, F., et al. (2018). Constraining Instantaneous Fluxes and Integrated Compositions of Fluvially Discharged Organic Matter. *Geochemistry, Geophysics, Geosystems*, *19*(8), 2453–2462. https://doi.org/10.1029/2018GC007539

Galy, V., Beyssac, O., France-Lanord, C., & Eglinton, T. (2008). Recycling of Graphite During Himalayan Erosion: A Geological Stabilization of Carbon in the Crust. *Science*, *322*(5903), 943–946. https://doi.org/10.1126/science.1161408
